# Comparison of Interobserver Reliability between Junior and Senior Resident in Assessment of Developmental Dysplasia of The Hip Severity using Tonnis and International Hip Dysplasia Institute Radiological Classification

**DOI:** 10.5704/MOJ.1911.010

**Published:** 2019-11

**Authors:** YD Ismiarto, P Agradi, ZN Helmi

**Affiliations:** Department of Orthopaedics and Traumatology, Padjadjaran University, Bandung, Indonesia; * Department of Orthopaedic Surgery, Lambung Mangkurat University, Banjarmasin, Indonesia

**Keywords:** DDH, IHDI classification, Tonnis classification

## Abstract

**Introduction:** The radiographic classification for developmental dysplasia of hip to quantify the severity of disease consist of Tonnis and International Hip Dysplasia Institute (IHDI) classification. The Ossification center of the femoral head in DDH patient more than six months is still vague or eccentric, so the reliability of both classifications is still in question and especially is influenced by the experience of the observer. This study aims to test and compare interobserver reliability in evaluation of DDH patients using IHDI and Tonnis classification assessed by senior and junior orthopaedic residents which had different degree of experience.

**Materials and Methods:** This study used retrospective analysis of pelvic supine AP view radiograph of DDH patients from 2014 to 2017. All three observer groups analysed the pelvis radiographs using Tonnis and IHDI classification. Inter and intra-observer reliability was measured by Cohen’s and Fleiss Kappa method, respectively. **Results:** The Fleiss Kappa value for 15 radiographs of DDH patients assessed by senior residents using Tonnis and IHDI classification are 0.715 and 0.832 and result of Fleiss Kappa value assessed by junior residents are 0.577 and 0.845, respectively. Intra-observer reliability for Tonnis classification was lower in junior group compared to other two groups but showed almost perfect value in all groups for IHDI classification.

**Conclusion:** Significantly different results were noted between junior and senior residents in assessing DDH severity, with higher diagnostic reliability in senior residents compared to junior residents. In general, junior resident has less clinical experiences in many aspects in comparison with the seniors.

## Introduction

Developmental dysplasia of hip (DDH) is an anatomical abnormality of the hip joint characterised by the head of the femur not having congruence with the os acetabulum. The incidence of DDH per 1000 live births has a significant variability in each racial group based on a specific geographical location and ranges from 0.06 in the African race to 76.1 per 1000 live births in the Native American race. In this disease there is a dominance in the left hip (64%) and the unilateral side (63.4%)^1^. Increased risk of DDH is found in the first female child, clubfoot abnormalities, and breech birth^2^. Diagnosis of DDH in the early period of infant is mainly dependent on clinical examination and hip ultrasonography. Ultrasonography of the hip joint is used for DDH screening in the first 4-6 months of life, but in older patients with DDH it is done using clinical and radiograph examination^3, 4^. Operative management in DDH patients requires several complicated and complex surgical procedures because there are pathological changes in the shape of bones, muscles, and tendons. A good pre-operative preparation and evaluation will determine the choice of type of surgical procedure to get the optimal results. For good surgical preparation, orthopedic experts need radiological classification to determine the severity of DDH^5, 6^.

Currently, there are several classifications used to assess the severity of DDH disease based on radiological examinations such as Severin classification, classification of Graf with sonography, Tonnis, and IHDI. From these classifications, there are two classifications that are most often used in assessing the severity of DDH using the anteroposterior pelvic radiograph, the Tonnis and IHDI classification^7, 8^. Assessment of the severity of DDH using the Tonnis method depends on the ossification center of the femur cap as the center of assessment and in some DDH patients, femoral capillary ossification appears late so that it appears unclear on the radiograph. This limitation can be a problem in assessing the degree of severity in DDH patients so it becomes more difficult and irrelevant. The limitations of the Tonnis method lead to the formation of a new method in assessing the severity of DDH, i.e. by the IHDI method.

The IHDI classification uses the midpoint on the proximal metaphysis of femur as the reference point so that it can be used to assess the severity of DDH of children in all age ranges^8^. This method is a new method which developed by the International Hip Dysplasia Institute (IHDI) and can be used to assess radiograph DDH patients with unclear ossification centers and eccentric locations^8-10^. A research by Narayanan in 2015 showed that the IHDI classification had better interobserver reliability than the Tonnis classification, but all assessors in the study were performed by an experienced paediatrics orthopedics^10^. Other studies also suggest that there are differences in the value of radiological classification reliability in other orthopedic cases assessed by observers with different experiences^11, 12^. Until now there has been no research on the reliability of the Tonnis and IHDI classifications that assessed by observers with different level of experiences. This study aimed to assess the reliability of the Tonnis method and the IHDI method in the assessment of pelvic radiograph of DDH patients assessed by observers with different levels of experience, namely senior orthopedic residents and junior orthopedic residents. The hypothesis is that the interobserver reliability value for both classifications will be higher in the senior group than junior group.

## Materials and Methods

To assess the reliability of Tonnis and IHDI radiological classification, we used total sampling methods digital anteroposterior (AP) pre-operative pelvic radiograph DDH patients who came to the Orthopedic and Traumatology outpatient clinic in 2014-2017 with inclusion criteria were DDH patients with an age range of 6-48 months, and femoral head ossification center is visible on the AP radiograph pelvis and exclusion criteria were the ossification center is not visible in the AP pelvic radiograph (patient age is less than six months), AP pelvic radiograph in DDH patients who have received previous surgery and a pelvic radiograph that does not meet the anteroposterior view requirements (the iliac crest appears asymmetrical, the obturator foramen, and the coccyx is not located in the middle when compared to the symphysis pubis).

**Fig. 1:**
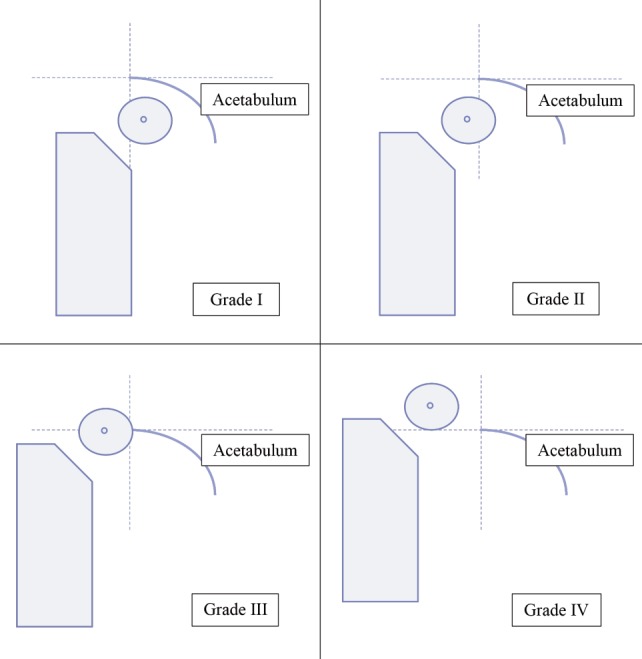
Schematic grading of DDH Severity based on the Tonnis classification.

After obtaining ethical approval from the hospital research ethics committee, number LB.04.01/A05/EC/380/XII/2017, a study was carried out by assessing the posterior anterior pelvic (AP) DDH patients using two radiological classification methods namely the Tonnis classification and IHDI classification. Tonnis classification used the Hilgenreiner (H-line) line, the line that connects the two bilateral triradiate cartilages; the Perkins line (P-line) which is a line drawn from the superolateral boundary of the acetabulum perpendicular to H-line. Based on those lines, the Tonnis classification divides the DDH severity into 4 grade (Fig. 1), which are Grade I: the ossification center of the femoral head is located medially from the Perkins line, Grade II: the ossification center of the femoral head is located laterally from the Perkins line, Grade III: the femoral head ossification center is located laterally and one level with the Perkins line, Grade IV: The ossification center of the femoral head is located next to the superolateral from the Perkins line.

**Fig. 2:**
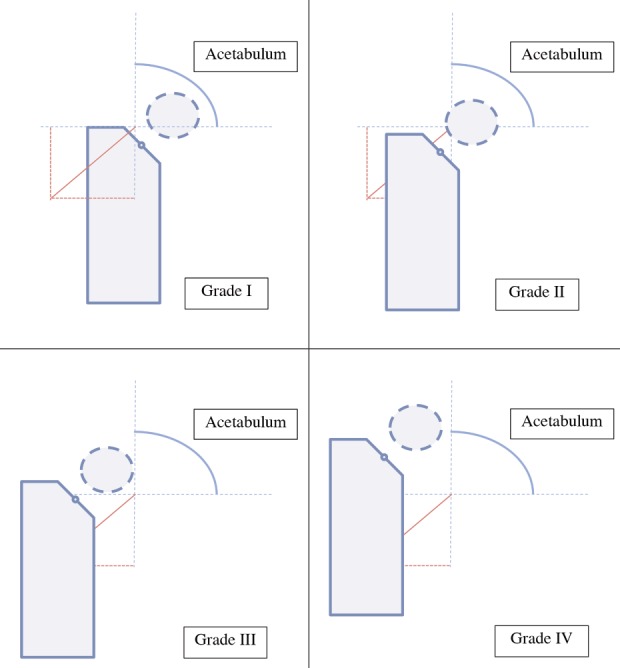
Schematic grading of DDH Severity based on IHDI classification.

The IHDI classification method uses the Hilgenreiner (H-line) reference line and the Perkins line (P-line) similar to the Tonnis classification in determining the grading of the severity of DDH patients. In the IHDI classification, there is an additional reference point, namely the D line (D-line), a 45-degree diagonal line drawn from the intersection between P-line and H-line, and point H (H-point) that is the center in the proximal part of the metaphysical femur. This IHDI classification (Fig. 2) also divides DDH severity into 4 grade, which are Grade I: Point H is located medially from the Perkins line, Grade II: Point H is located lateral to the Perkins line and medial to line D, Grade III: The H point is located lateral to the D line and inferior to the Hilgenreiner line, Grade IV: Point H is located next to the superior of the Hilgenreiner line.

The digital radiograph pelvis AP samples were selected by authors and co-authors who was not involved in assessment of the radiograph samples and then analysed by 12 independent assessors divided into 3 groups, group A consisted of 4 senior orthopaedic residents who had completed all rotations in the paediatric orthopaedic sub-section, group B consisted of 4 junior orthopaedic residents who had never take part in any rotation in the paediatric orthopaedic sub-section, and group C consisted of 4 staff surgeons as a control group and did not involved in any care of the patients. All assessors analyse the AP pelvic radiograph using the Tonnis and IHDI classifications. Before making a trial assessment, each assessor in groups will be provided and explained by the authors with a piece of paper containing a definition of the severity of each classification to reduce bias due to errors in interpretation. Authors also demonstrated the assessment of radiograph step by step to classify the severity using both classifications on sample radiograph that not included in the samples. Patient radiograph samples will also be displayed randomly and anonymously to reduce recall bias or memory. Assessment of radiographs samples is performed twice with 10 days interval between each assessment trial.

Statistical analysis was performed using SPSS version 23.0 software, interobserver reliability was measured using the Fleiss kappa method, and intra-observer reliability was measured by Cohen’s kappa. The Fleiss and Cohen’s Kappa interpretation used the standard recommended by Landis and Koch as seen in (Table I)^13^.

**Table I:** Weighted Kappa Score based on Landis and Koch^13^

Weighted Kappa Score	Interpretation
< 0 Poor	
0.01-0.2	Slight
0.21-0.40	Fair
0.41-0.60	Moderate
0.61-0.80	Substantial
0.81-1.00	Almost perfect

## Results

In this study, total of 18 pre-operative radiograph samples were obtained for DDH patients with age range between 6-42 months, there were 15 radiograph samples that met the inclusion criteria as above but there were 3 radiograph samples which did not meet the inclusion criteria. This study was continued by analysing 15 radiograph DDH patients using the Tonnis and IHDI classifications by three assessment groups consisting of 4 junior, 4 senior orthopaedic residents, and 4 staff surgeons, respectively. The interobserver and intra-observer reliability score for the Tonnis and IHDI classifications are listed in (Table II, III), respectively.

**Table II:** Interobserver reliability for Tonnis and IHDI classifications in all groups

Assessor Group	Tonnis (n=15)		IHDI (n=15)	
	Fleiss Kappa	95%CI	Fleiss Kappa	95%CI
Senior Resident	0.715	0.569-0.861	0.832	0.685-0.979
Junior Resident	0.577	0.430-0.724	0.845	0.697-0.993
Staff Surgeon	0.732	0.586-0.879	0.899	0.753-1.046

**Table III:** Frequency of the symptoms

Assessor Group	Tonnis (n=15) Cohen’s Kappa	IHDI (n=15) Cohen’s Kappa
Senior Resident		
1	0.898	0.797
2	0.899	1.000
3	0.900	1.000
4	0.898	1.000
Junior Resident		
1	0.797	1.000
2	0.789	1.000
3	0.803	0.899
4	0.783	1.000
Staff Surgeon		
1	1.000	1.000
2	0.800	1.000
3	0.704	0.900
4	1.000	1.000

Based on Landis and Koch's standards, the Fleiss Kappa score in Tonnis classification shows the moderate value (0.577) for the junior assessor and substantial value for the senior (0.715) and staff surgeon (0.732) assessor. This shows a difference value in interobserver reliability using the Tonnis classification between junior and senior assessment groups that have different levels of experience. But, same substantial value of interobserver reliability was found in the senior group (0.715) and staff group (0.732). Based on Landis and Koch's standards, interobserver reliability in IHDI classification assessed by the three observer groups showed values that were classified as Almost Perfect. The IHDI classification has a Fleiss Kappa score with a value of 0.832 and 0.845 in the junior and senior assessment groups respectively and 0.899 in the staff surgeons group. In (Table II), it can be seen a comparison that the Tonnis classification has Fleiss Kappa score lower than the IHDI classification in the junior, senior and staff surgeons assessment groups. This shows that the reliability of the IHDI classification is better than the Tonnis classification in assessing the severity of DDH disease.

As seen on (Table III), according to Landis and Koch, the intra-observer reliability for IHDI classification showed almost perfect score in all groups. The different result showed on Tonnis classification, the intra-observer reliability in junior group was lower than other two groups. The junior resident group has average substantial value but senior and staff surgeons groups has almost perfect value for intra-observer reliability.

## Discussion

**Fig. 3:**
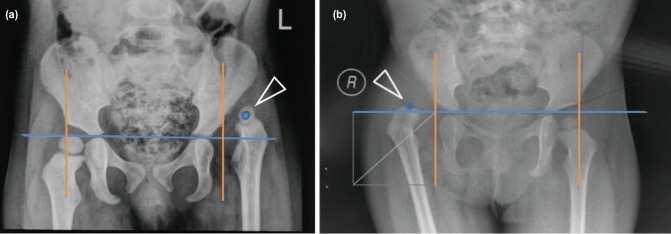
(a) One example of AP pelvic radiograph with grade 4 Tonnis classification and (b) grade 4 IHDI classification. From the radiograph it can be seen that the ossific nucleus of the right femoral head is not visible on the radiograph.

From the results, it showed that in accordance with the hypothesis, the interobserver reliability score in junior group was lower than senior group. The senior group result was same with the staff surgeons group as control group. In this study there were significant differences in the reliability test of the Tonnis classification in the junior and senior assessor groups. The junior assessor has a Fleiss Kappa Moderate value (0.577) and the Fleiss Kappa value in the senior assessor has a Substantial value (0.715). This shows that the interobserver reliability of the Tonnis classification in the senior assessment group is better than the junior assessor. But in the senior and staff group, the interobserver reliability had the same result, which is Substantial value. This value difference occurs because the Tonnis classification has difficulties in assessing the pelvic radiograph with the os femur head is not very clear, and this difference is also influenced by the different levels of experience between junior assessors and senior assessors, giving rise to assumptions in assessing the femoral os position against Perkin's line, as shown in (Fig. 3). According to research by Narayanan also revealed that in the Tonnis classification it was found that there were difficulties in assessing the severity of DDH in 10 pelvic radiograph with vague or unclear femoral os^10^. In DDH patients who come to the clinic late or when the patient is able to walk (late presenting), on pelvic AP radiograph examination it is found that the femoral os has not meet met perfect ossification so the clinician has difficulty in determine the severity of DDH disease^7^.

Unlike the case assessment with the Tonnis classification, the three assessment groups were seen that the interobserver reliability of the IHDI classification was classified as Almost Perfect. The results of the study showed that the Fleiss kappa value in the IHDI classification in all groups was classified as almost perfect (0.81-1.00), and the Fleiss Kappa value is greater when compared to the Tonnis classification which has a moderate value (0.577) for the junior groups and a substantial value (0.715) for Senior groups. This occurs because the IHDI classification provides more benefit and ease in assessing the severity of DDH disease, specifically by using a proximal femoral metaphysis H point that appears clearly as a reference in the assessment. From the Fleiss Kappa results it can be seen that the IHDI classification has better interobserver reliability compared to the Tonnis classification so that the IHDI classification is more reliable in assessing the severity of DDH patients even though it is carried out by assessors with different levels of experience. This study is also in accordance with the research conducted by Miao and Narayanan who also suggested that the IHDI classification had better interobserver reliability compared to the Tonnis classification, but the assessors in the study were orthopaedic experts who had the same level of experience^9, 10^.

Differences of radiologic criteria may result in higher interobserver reliability in IHDI classification compared to Tonnis classification while assessing DDH. The IHDI classification judges the superior margin of proximal femoral metaphysis as a line whilst the Tonnis classification judges the proximal femoral ossific nucleus as a circle or quasi-circle. The latter system is difficult to apply in blurry radiological images. The H-point in IHDI classification point are defined in the midpoint in a metaphysis margin line; with Tonnis classification determines the H-point as the circle’s center. Thus, it is much easier to judge a line and midpoint compared to judging a circle’s boundary and center. The final reason would be the usage of D-line in IHDI classification that may increase the accuracy of evaluation of DDH due to division of equal parts of lower outer quadrants^9, 10^.

Based on Landis and Koch in (Table I), intra-observer reliability score when using Tonnis classification was better in senior and staff surgeon groups than the junior group. Contrary to Tonnis classification, intra-observer reliability by using the IHDI classification has almost perfect value in all groups (Table III). This results identified that there was influence of different level of experience on the intra-observer reliability score in our study. The Tonnis classification has several disadvantages, such as it relies on the ossification center of the femur so that in DDH patients between the ages of 6-18 months with a center of ossification of the femur caput that has not been clearly seen and its eccentric location will cause difficulties in determining the severity^10^. This study had two disadvantages. Firstly, recall bias can still occur because most radiograph images have has a picture of almost the same severity. Secondly, the sample size was small because this study used a retrospective observational design and was carried out in only one education center.

## Conclusion

The inter and intra-observer reliability of Tonnis classification has significant difference between junior and senior resident. This is due to difficulty in judgments using Tonnis classification that are influenced by different degrees of experience. The IHDI classification system may be subjectively easier to use compared to the Tonnis classification in assessing the severity of DDH due to its ease in assessing radiological pictures of the hip. Significantly different results were noted between junior and senior residents in assessing DDH severity, with higher reliability in senior residents compared to junior residents. In general, junior resident had less clinical experiences in many aspects in comparison with the seniors.
